# Bortezomib-Induced Reticular Eruption in Patient with Multiple Myeloma

**DOI:** 10.3390/dermatopathology10030031

**Published:** 2023-07-21

**Authors:** Joseph Han, Shayan Owji, Aneesh Agarwal, Samir Kamat, Yen Luu, Adnan Mubasher, George Niedt, Chloe Ray, Hearn Jay Cho, Nicholas Gulati, Angela Lamb

**Affiliations:** 1 Department of Dermatology, Icahn School of Medicine at Mount Sinai, New York, NY 10029, USA; 2 School of Medicine, University of Missouri-Kansas City, Kansas City, MO 64108, USA; 3 Tisch Cancer Institute, Icahn School of Medicine at Mount Sinai, New York, NY 10029, USA

**Keywords:** bortezomib, cutaneous drug eruption, multiple myeloma, reticular

## Abstract

Bortezomib is the first proteasome inhibitor to treat a variety of malignancies and is currently part of the standard of care regimen for the initial treatment of patients with newly diagnosed multiple myeloma. While bortezomib is generally well tolerated, it has been associated with various side effects, which have limited its use in some patients. Here, we describe a unique case with histological confirmation of a reticular eruption that appeared at the site of a subcutaneous administration of bortezomib in a 62-year-old male who was newly diagnosed with IgG kappa multiple myeloma. A skin biopsy was performed, which revealed superficial perivascular dermatitis predominantly composed of lymphocytes with rare eosinophils. The patient was successfully treated with betamethasone dipropionate 0.05% cream. When consulted, dermatologists should advise the oncology team of multiple myeloma patients treated with bortezomib to maintain a high threshold before discontinuing the drug when a patient experiences an atypical, reticular rash following subcutaneous administration. Additionally, potent topical corticosteroids, such as betamethasone dipropionate 0.05% cream, should be considered in managing the cutaneous reticular eruptions related to bortezomib administration, in order to maintain an optimal treatment regimen for patients with multiple myeloma.

## 1. Introduction

Bortezomib is the first proteasome inhibitor to treat a variety of malignancies and is currently part of the standard of care regimen for the initial treatment of patients with newly diagnosed multiple myeloma [1]. While bortezomib is generally well tolerated, it has been associated with various side effects which have limited its use in some patients. Cutaneous adverse reactions to subcutaneous bortezomib administration have been reported in 10–24% of patients and are generally present as cutaneous nodules, plaques, or morbilliform erythema [2,3]. Additionally, bortezomib has also been associated with cutaneous vasculitis, a “folliculitis-like” rash, and neutrophilic dermatosis [4,5]. While unconfirmed, it has been proposed that the mechanism for some of these cutaneous reactions involves drug-induced inflammatory cytokine amplification [6]. Bortezomib-related skin reactions generally present after multiple treatment cycles, and though they will often resolve quickly following antihistamine and corticosteroid treatment or within a week following the last dose without pharmacological intervention, recurrence can be a challenge in subsequent treatment cycles [7]. Here, we describe a unique case, with histological confirmation, of a reticular eruption that appeared at the site of a subcutaneous administration of bortezomib.

## 2. Case Report

The patient is a 62-year-old male who was newly diagnosed with IgG kappa multiple myeloma. Upon starting treatment a month following the initial diagnosis, the patient’s first treatment cycle included 1.3 mg/m^2^ of bortezomib on days 1, 8, and 15; 25 mg of lenalidomide daily; and 40 mg of dexamethasone once a week. Each medication was prescribed as part of a three-week treatment cycle, with a one-week rest period in between cycles. The patient was also started on 81 mg of aspirin daily for deep vein thrombosis prophylaxis and 400 mg of acyclovir daily for shingles prophylaxis.

**Figure 1:**
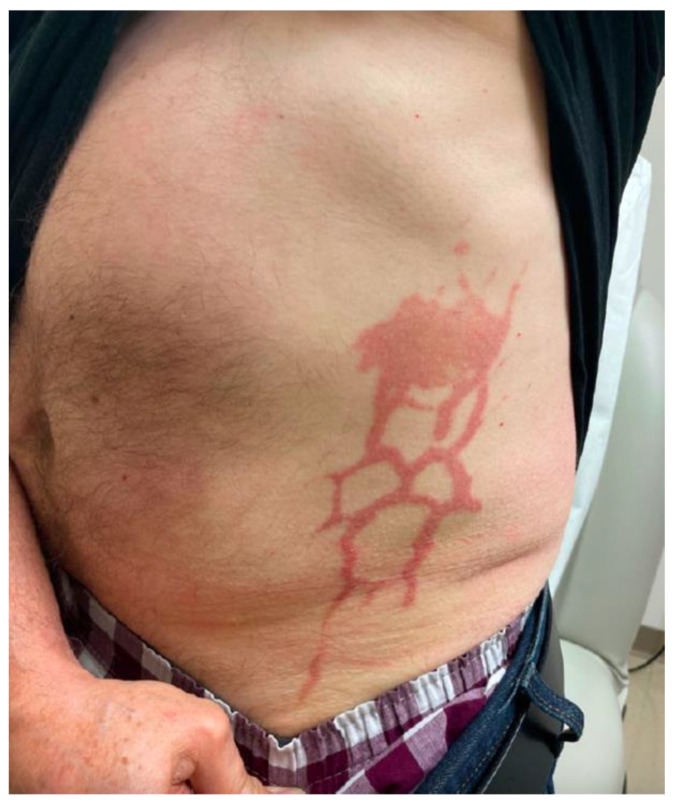
Reticular eruption of the left flank following a subcutaneous injection of bortezomib in the patient’s third cycle of treatment.

One day, after the first injection of bortezomib in the third cycle of his treatment, the patient experienced a pronounced erythematous and pruritic plaque at the injection site on the left flank, with a strikingly reticular pattern inferiorly mirroring a vascular distribution (Figure 1). In his first two cycles, the patient only experienced a much more localized reaction without a reticular pattern, which self-resolved within a few days.

**Figure 2:**
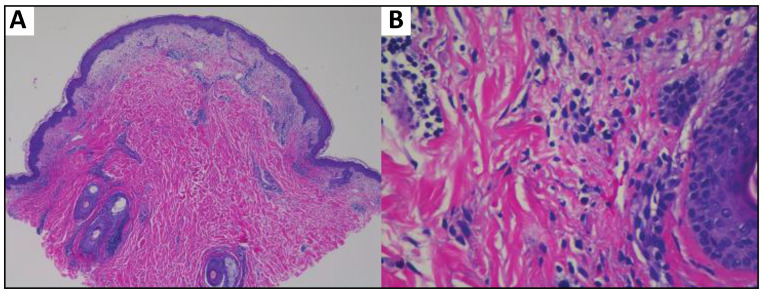
Superficial perivascular dermatitis with lymphocytes and eosinophils consistent with a drug eruption. (**A**) Low magnification (4×), (**B**) high magnification (40×).

The oncology team held all treatment until a dermatology evaluation was performed because of this rash. The patient was instructed to use betamethasone dipropionate 0.05% cream by his dermatologist and a skin biopsy was performed eight days following the appearance of the rash, which revealed superficial perivascular dermatitis predominantly composed of lymphocytes with rare eosinophils (Figure 2).

**Figure 3:**
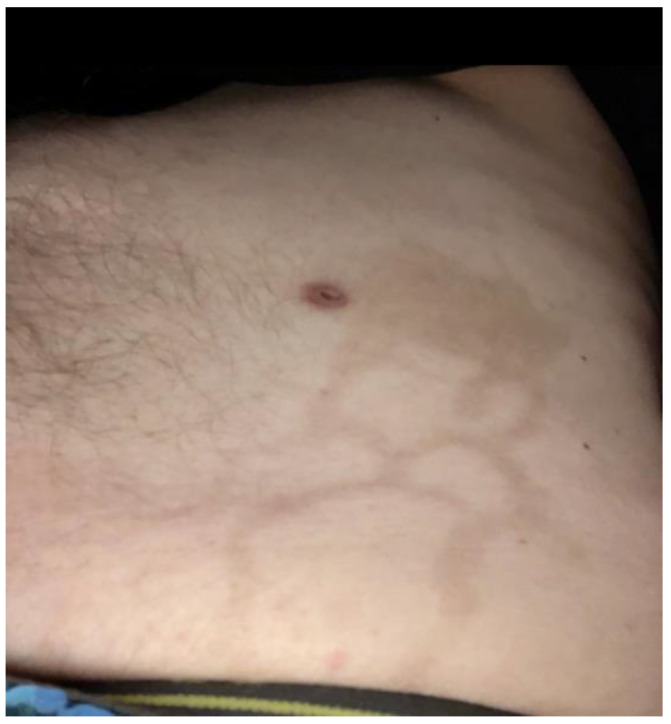
Post-inflammatory hyperpigmentation at the site of the reticular eruption of the left flank following treatment with betamethasone dipropionate 0.05% cream.

With the application of betamethasone dipropionate 0.05% cream once daily, the pruritus and erythema resolved after two to three weeks. After seven weeks, only post-inflammatory hyperpigmentation was observed at the site (Figure 3). The patient did not have any other adverse events and treatment was restarted five days after the dermatology appointment, with no further recurrence of this rash.

## 3. Discussion

While there has been a report of a spider-like cutaneous reaction from bortezomib [8], this is, to our knowledge, the first report of a subcutaneous bortezomib-induced reticular drug eruption at the site of the injection with histological confirmation. Perivascular lymphocytic reactionary side effects have been found with bortezomib use, though previous reports describe eruptions as erythematous with multiple nodules, which was not observed in our patient who also had eosinophil presence [2,9]. Histologic differential diagnoses for superficial perivascular dermatoses with lymphoeosinophilic infiltrate include atopic and chronic allergic/contact dermatitis, scabies, and drug reactions [10]. The rash’s remarkable reticular distribution is reminiscent of supravenous serpentine eruption or hyperpigmentation often seen with the intravenous administration of various chemotherapy agents, thought to be due to the extravasation of the cytotoxic agent after endothelial cell damage [11]. In cases of these reactions, which are frequently characterized by dermal perivascular lymphocytic infiltrates, the reaction is often initially erythematous, followed by residual hyperpigmentation [12,13]. However, this patient was only receiving subcutaneous bortezomib injections and he only experienced this rash after 1 of 10 injections, possibly reflecting the unintended entry of the agent into a larger vessel.

While systemic corticosteroids, such as prednisone, have been used for cutaneous reactions to bortezomib, this treatment may lead to an altered immune function as an undesirable side effect [7,14,15]. Therefore, we recommend treating any associated pruritus with antihistamines or topical corticosteroids before considering systemic therapy for cutaneous reactions. In order to avoid altering the pharmacokinetic properties of bortezomib, it has been suggested that interventions such as antihistamines or topical corticosteroids not be used within four hours of its administration [16].

The subcutaneous route is often preferred due to being less invasive and more comfortable for the patient. However, in cases where cutaneous reactions are severe or recurring following this method, it may be worthwhile to consider alternate approaches for bortezomib administration, such as the intravenous route; in fact, the literature reports that both subcutaneous and intravenous administration of bortezomib have similar response rates, efficacies, toxicity profiles, and rates of adverse events [17]. As a result, if there is concern surrounding skin reactions, the intravenous route may help more completely avoid cutaneous side effects and should be given consideration, especially in patients with an established port or line. However, since there is an increased risk of peripheral neuropathy with intravenous bortezomib dosing, the presence of existing risk factors, such as baseline peripheral neuropathy, diabetes, and previous neurotoxin exposure, should be assessed [18]. Furthermore, intravenous injection may also avoid the potential complication of tissue injury from the accumulation of unabsorbed medication associated with a subcutaneous injection [19].

Of the prescribed subcutaneous bortezomib protocol, our patient missed one dose as a result of the rash, and his bortezomib treatment was resumed after the dermatology appointment. While no negative implications were evident from missing a dose in our case, Loke et al. demonstrated better overall survival in multiple myeloma patients receiving higher doses (70 mg or a greater total dose) of bortezomib [20]. Therefore, in more aggressive cases of myeloma, it may be advisable to avoid missing doses of bortezomib as a result of manageable cutaneous reactions. In addition, we recommend that oncology teams maintain a higher threshold before discontinuing bortezomib when a patient experiences an atypical, reticular rash following subcutaneous administration of the drug. However, in our case, the patient experienced an atypical injection site reaction from bortezomib that a dermatologist was called upon to evaluate and manage. The patient was successfully treated with potent topical steroids and was able to restart his bortezomib without any adverse effects.

## 4. Conclusions

Overall, when consulted, dermatologists should advise the oncology team of multiple myeloma patients treated with bortezomib to maintain a high threshold before discontinuing the drug when a patient experiences an atypical reticular eruption following subcutaneous administration. Potent topical corticosteroids such as betamethasone dipropionate 0.05% should be considered in managing cutaneous reticular eruptions related to bortezomib administration in order to maintain an optimal treatment regimen for patients with multiple myeloma.

## Data Availability

No new data were created or analyzed in this study. Data sharing is not applicable to this article.
